# Advances in the Structural Basis of GluN2A-Selective Negative Allosteric Modulators

**DOI:** 10.1021/acsmedchemlett.5c00226

**Published:** 2025-05-09

**Authors:** Yinlong Li, Hongjie Yuan, Steven H. Liang

**Affiliations:** † Department of Radiology and Imaging Sciences, 1371 Emory University, 1364 Clifton Road, Atlanta, Georgia 30322, United States; ‡ Department of Pharmacology and Chemical Biology, Emory University School of Medicine, Atlanta, Georgia 30322, United States

**Keywords:** NMDARs, GluN2A subunit, Negative allosteric modulators (NAMs), Structure−activity relationship (SAR), CNS disorder

## Abstract

*N*-Methyl-d-aspartate receptors (NMDARs) are ligand-gated ion channels that play a critical role in synaptic plasticity, learning, and memory in the central nervous system (CNS). These receptors assemble into functionally diverse complexes composed of GluN1, GluN2, and GluN3 subunits. Among them, GluN1/GluN2A is expressed predominantly in adult brain, and its dysfunction has been implicated in various neuropathological conditions. Recent advances have identified several GluN2A-targeted agents, including highly selective negative allosteric modulators (NAMs). Structure–activity relationship (SAR) studies of existing agents have provided valuable insights into structural modifications that enhance pharmacological properties, offering promising avenues for therapeutic development.

## Graphical abstract

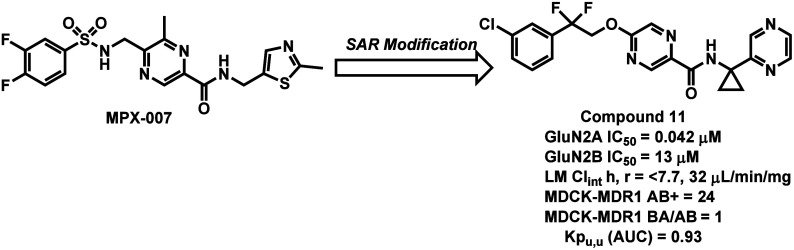

## Introduction

*N*-Methyl-d-aspartate receptors (NMDARs) are a subtype of ionotropic glutamate receptors (iGluRs) that play a crucial role in functional and structural plasticity in the central nervous system (CNS).^1–5^ As ligand-gated ion channels, NMDARs are activated by the excitatory neurotransmitters to mediate the passage of calcium (Ca^2+^), sodium (Na^+^), and potassium (K^+^) ions across the neuronal membrane.^6^ NMDARs are voltage-dependent blocked by extracellular magnesium (Mg^2+^) under resting membrane potential. However, this blockage can be relieved when the two coagonists, glycine (or d-serine) and l-glutamate simultaneously bind to GluN1 and GluN2 subunits, respectively, gating the channel pore open, allowing the influx of Ca^2+^ and Na^+^ through the plasma membrane (Figure 1).^7,8^ This unique gating mechanism triggers serial intracellular signaling cascades, including long-term potentiation (LTP) and long-term depression (LTD), which are fundamental for learning and memory.^9,10^ Functional NMDARs are heterotetrameric complexes, consisting of two obligatory GluN1 subunits and two GluN2 (A-D) and/or GluN3 (A-B) subunits.^11,12^ Among these, GluN2A-containing NMDARs are highly expressed in the brain, especially in the cortex, hippocampus and striatum regions strongly associated with both acute and chronic neurodegenerative diseases.^13,14^ The critical role of the GluN2A subunit is clearly evidenced by its involvement in a wide range of neurological and neuropsychiatric disorders, such as autism, depression, epilepsy, intellectual disability, language/speech disorders, movement disorders, and schizophrenia.^15–18^ Consequently, therapeutic strategies targeting GluN2A-containing NMDARs with specialized agents have garnered significant interest.^19–22^

**Figure 1:**
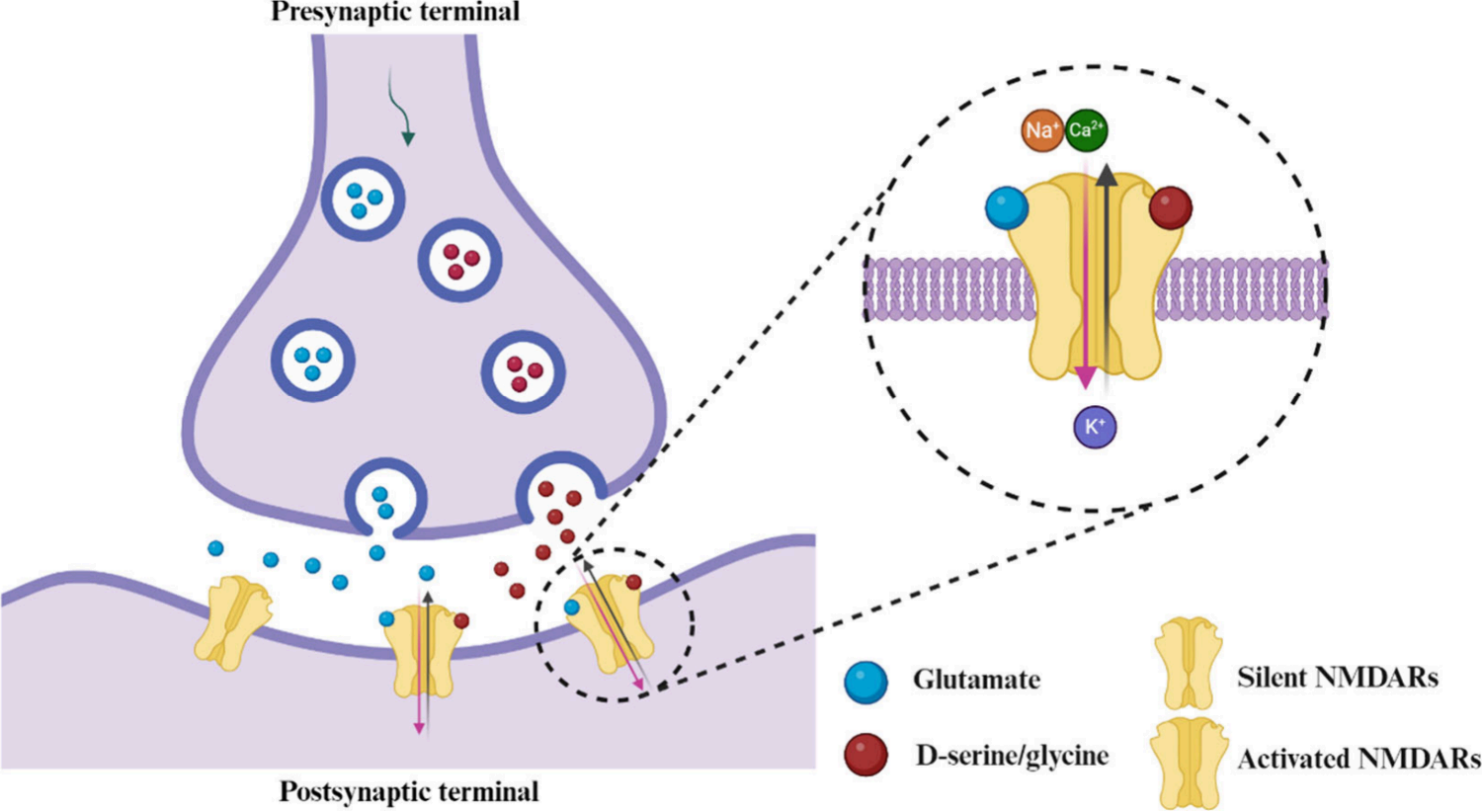
Location and function of NMDARs in synaptic plasticity. This figure was created using BioRender (BioRender.com) under a BioRender publication license.

Exogenous regulators such as agonists and antagonists act on the orthosteric binding site of GluN2A, either mimicking or inhibiting the action of endogenous ligands.^23^ However, the high conservation of orthosteric binding sites across NMDAR subunits poses a significant challenge in developing GluN2A-selective ligands. In contrast, negative allosteric modulators (NAMs) and positive allosteric modulators (PAMs) target allosteric sites, providing advantages such as higher subunit selectivity.^23,24^ Recent efforts have focused on discovering selective modulators for GluN2A, yet suitable ligands remain lacking and no GluN2A-selective ligands have been approved for clinical use. Based on sulfonamide derivatives, TCN-201, the prototype of GluN2A-selective NAM was identified through a high throughput screening (HTS) method in 2010,^25^ and negatively modulates glycine affinity rather than competing with glycine-binding sites in the GluN1 subunit directly.^26^ TCN-201 exhibited submicromolar affinity toward GluN2A with high selectivity over GluN2B and GluN2D, but its modest potency and poor solubility limit its biological application. Systematic structural modification of the benzenesulfonamide scaffold in TCN-201 led to the identification of MPX-004 and MPX-007 with showed improved potency and solubility (Figure 2).^27^ However, the neuropharmacological applications of these compounds is hampered by their high polar surface area (TPSA: 151 Å^2^) and poor blood-brain barrier (BBB) permeability.

**Figure 2:**
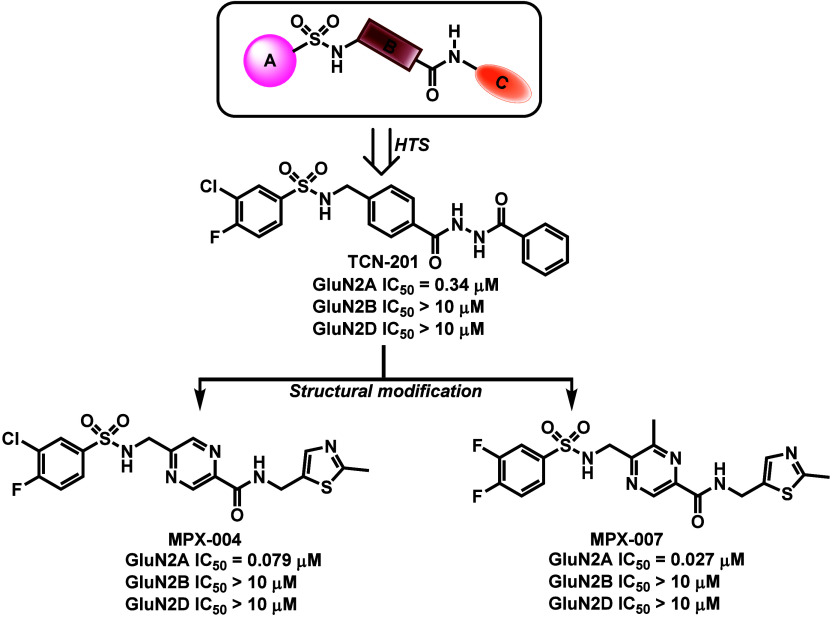
Representative GluN2A-selective NAMs. The reported IC_50_ values were obtained by measuring the inhibition of Ca^2+^ responses mediated by GluN2A, GluN2B and GluN2D coexpressed with GluN1 in human embryonic kidney (HEK) cells. The data was adapted from ref 27. Copyright 2016 Volkmann et al.

Crystallography studies have revealed that the binding site of TCN-201 and MPX-004/007 is located at the dimer interface between the GluN1/2A ligand binding domains (LBDs). Structure–activity relationship (SAR) analysis indicates that π-π stacking interactions occur between aromatic rings A and B (Figure 2) within the NAM binding pocket. The 3-halogen substituent on ring A is essential for maintaining potency, while rings B and C are more flexible and can accommodate heteroaryl moieties. Recently, Bischoff et al. conducted further SAR exploration of MPX-004/007 by optimizing the linker region between the distal halogenated aromatic ring A and the central pyrazine ring B (Figure 3).^28^ One major challenge was the high P-gp efflux ratio (BA/AB = 37) of MPX-007, potentially caused by its high polar surface area. To address this, the authors replaced the 2-methylthiazole ring with pyridine or pyrazine to reduce P-gp efflux. Additionally, to reduce the number of hydrogen bond donors, the -NHCH_2_- group in the sulfonamide linker was replaced with -CH_2_O-. The newly designed compounds **1–3** demonstrated high inhibitory potency against GluN2A (IC_50_: 0.016 to 0.022 μM) and high selectivity over other subunits (IC_50_ ≥ 28 μM). However, these compounds exhibited metabolic instability in human and rat liver microsomes. Replacing the sulfone group with a benzylic CF_2_ group yielded compounds **10–12**, which showed a slight decrease in potency (IC_50_: 0.040 to 0.069 μM) but retained high selectivity. Of note, the metabolic stability in human and rat liver microsomes was significantly improved (Clint: 7.7–75 μL/min/mg). Furthermore, all modified compounds exhibited lower P-gp efflux ratios (BA/AB ≤ 6) compared to MPX-007 (BA/AB = 37), suggesting enhanced BBB permeability and greater potential for CNS applications.

**Figure 3:**
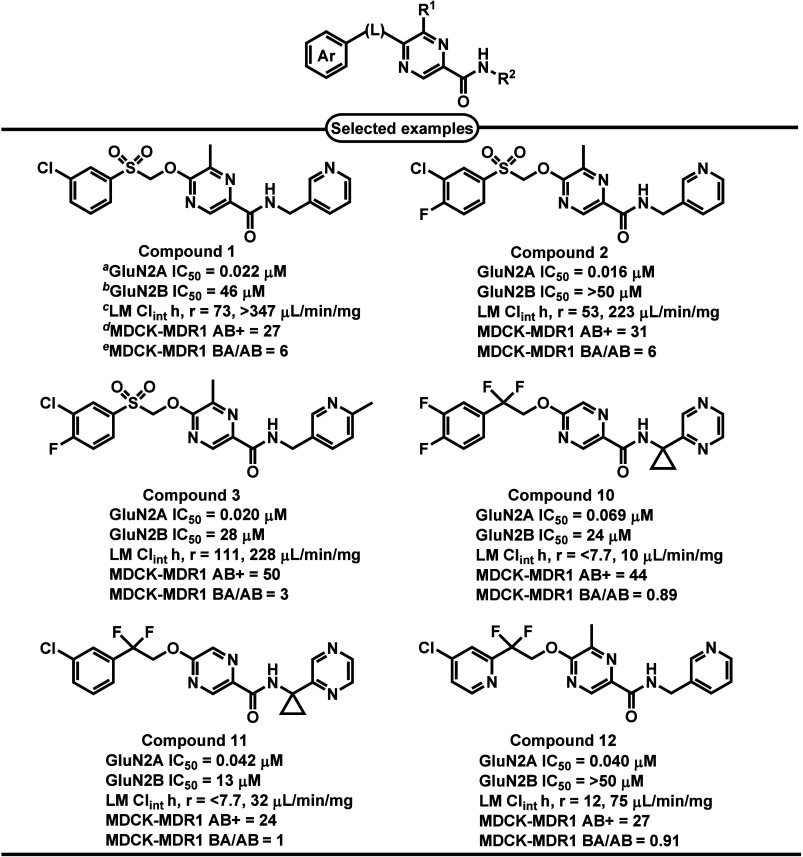
SAR optimization of MPX-004/007 scaffold. ^*a,b*^The IC_50_ values were determined by fluorometric calcium mobilization assays. ^*c*^Intrinsic clearance (Clint, μL/min/mg microsomal protein) was tested in human liver microsomes and rat liver microsomes. ^*d*^Apparent permeability (10^–6^ cm/s, A to B) was measured in MDCK-MDR1 cells. ^*e*^BA/AB = P-gp efflux ratio. The data was adapted from ref 28. Copyright 2025 American Chemical Society.

Another aim of the study was to evaluate target engagement of the designed compounds. The authors utilized tritium-labeled [^3^H]**1** to investigate whether receptor expression was conserved across species through in vitro autoradiography studies. As shown in Figure 4A–D, high uptake of [^3^H]**1** was observed in the hippocampus and cortex, a pattern conserved across mouse, rat, dog, and nonhuman primate (NHP) models. In GluN2A knockout (KO) mice, total binding levels were comparable to nonspecific binding observed when coincubation of cold compound **2**. The selectivity of [^3^H]**1** was further assessed by treatment with MPX-007 (a GluN2A inhibitor), Ro-25-6981 (a GluN2B inhibitor), and MK-801 (a pan-NMDAR inhibitor) (Figure 4E). The results demonstrated that only MPX-007 blocked the uptake of [^3^H]**1**, confirming its selectivity for GluN2A. Furthermore, GluN2A receptor occupancy was investigated in rats following an oral dose of compound **11** in an ex vivo study, revealing 42% receptor occupancy and good brain penetration (Figure 4F). Collectively, these findings indicate that the designed compounds are potent and selective GluN2A NAMs with improved drug-like properties.

**Figure 4:**
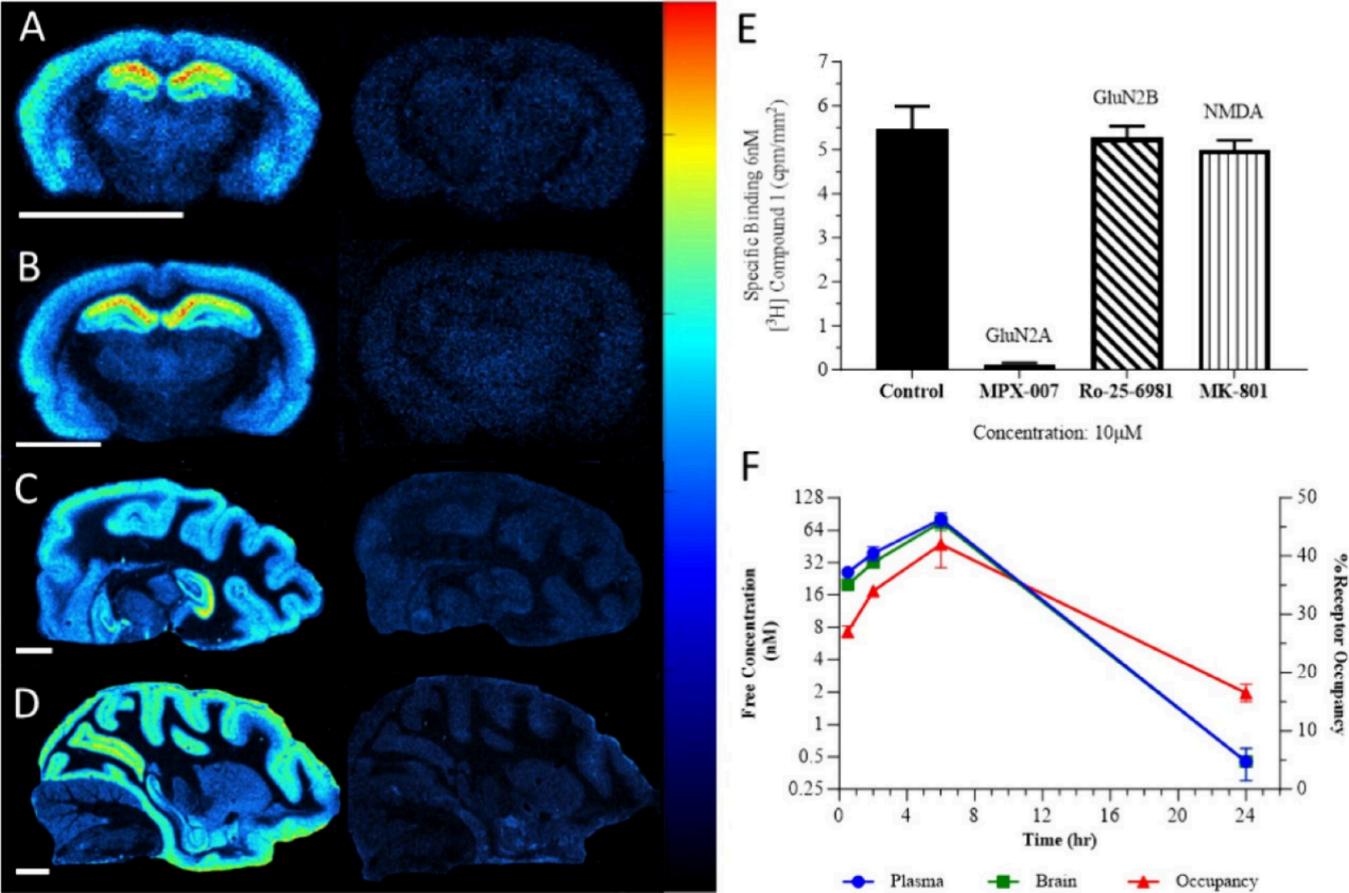
(A) In vitro autoradiography studies of ligand [^3^H]**1** in WT mouse (left) and GRIN2A KO tissue (right). (B–D) Autoradiograms of rat (B), dog (C), and NHP (D) tissues, with the left panel depicting total binding and the right panel showing nonspecific binding. (E) The selectivity of [^3^H]**1** was evaluated via in vitro autoradiography using known NMDA ligands: MPX-007 for GluN2A, Ro-25-6981 for GluN2B, and MK-801 for all NMDARs. (F) Compound **11** was administered at 25 mg/kg in rats, and GluN2A receptor occupancy, the unbound plasma and unbound brain concentrations was plotted vs time (*n* = 2 per time point ± SEM). The data was adapted from ref 28. Copyright 2025 American Chemical Society.

## Future Outlook

The development of GluN2A-selective NAMs holds great promise for advancing our understanding of NMDAR-mediated signaling and for therapeutic applications in neurological disorders. Significant progress has been made in the development of subunit-selective modulators for NMDARs, with several new binding sites identified. While various antagonists targeting NMDAR subunit have been discovered, the development of selective GluN2A agents remains limited. Starting with TCN-201 as the first-in-class GluN2A-selective NAM, analogs such as MPX-004 and MPX-007 were subsequently identified and showed improved potency. However, challenges persist in overcoming high P-gp efflux and poor metabolic stability. Future efforts should focus on structural modifications to enhance bioavailability, brain penetration, and metabolic stability while maintaining high selectivity.

Another major challenge in studying GluN2A in vivo is the lack of highly specific molecular probes such as positron emission tomography (PET) tracers. The development of radiolabeled GluN2A-selective PET ligands would enable noninvasive imaging of receptor distribution, occupancy, and dynamics in living subjects. This advancement could facilitate GluN2A-selective drug development by providing crucial pharmacokinetic and target engagement information, ultimately accelerating translational research.^29–32^
